# Co-delivery of phagocytosis checkpoint and STING agonist by a Trojan horse nanocapsule for orthotopic glioma immunotherapy

**DOI:** 10.7150/thno.73104

**Published:** 2022-07-18

**Authors:** Ying Zhou, Yuxin Guo, Lufei Chen, Xiaoli Zhang, Wei Wu, Zhimin Yang, Xuejie Li, Yuanzhuo Wang, Zhiyuan Hu, Zihua Wang

**Affiliations:** 1 Fujian Provincial Key Laboratory of Brain Aging and Neurodegenerative Diseases, School of Basic Medical Sciences, Fujian Medical University, Fuzhou, Fujian 350122, China.; 2 CAS Key Laboratory of Standardization and Measurement for Nanotechnology, CAS Key Laboratory for Biomedical Effects of Nanomaterials and Nanosafety, CAS Center for Excellence in Nanoscience, National Center for Nanoscience and Technology, Beijing 100190, China.; 3 School of Nanoscience and Technology, Sino-Danish College, University of Chinese Academy of Sciences, Beijing 100049, China.; 4 School of Chemical Engineering and Pharmacy, Wuhan Institute of Technology, Wuhan, 430205, China.

**Keywords:** CD47, glioblastoma, immunotherapy, tumor associated macrophages, phagocytosis

## Abstract

**Rationale:** Cancer immunotherapy has demonstrated significant antitumor activity in a variety of tumors; however, extensive infiltration of immunosuppressive tumor-associated macrophages (TAMs) in the glioblastoma (GBM) tumor microenvironment (TME) and the existence of the blood-brain barrier (BBB) might lead to failure of the checkpoint blockade therapy.

**Methods:** Herein, we have developed a smart “Trojan horse” BBB-permeable nanocapsule termed “NAcp@CD47” to deliver anti-CD47 antibodies and stimulator of interferon genes (STING) agonists into GBM tissues in a stealth-like manner to reshaped the immune microenvironment by switching the phenotype of microglia and macrophages.

Results: Both in vitro and in vivo studies demonstrate that NAcp@CD47 could effectively penetrate the BBB, increase the polarization of M1-phenotype TAMs, help reduce tumor immunosuppression, and inhibit the orthotopic GBM growth by phagocytosis of macrophages and microglia.

**Conclusions:** Our findings indicate that the well-designed NAcp@CD47 not only enhances the phagocytosis of cancer cells but also efficiently enhance antitumor immunogenicity and reverses immune suppression to convert uninflamed “cold” tumors into “hot” tumors.

## Introduction

Glioblastoma (GBM; World Health Organization grade IV astrocytoma) is the most common and aggressive type of primary malignant brain tumor in adults 1, 2. Over the past few decades, GBM has been regarded as one of the most lethal forms of human cancers. Despite advancements in the standard of care for newly diagnosed GBM, including surgical resection, radiotherapy, and chemotherapy, prognosis remains poor, and the median survival of patients is only 12-15 months 3-5. The poor outcomes of current treatments might be attributed to the following challenges: radioresistance and chemoresistance, diffuse infiltrative growth, presence of glioma stem cells, high rates of recurrence, intertumoral and intratumoral heterogeneity, poor drug blood-brain barrier (BBB) penetration, and lack of ideal preclinical models 2, 6. Thus, more innovative and advancing therapies for GBM are urgently needed. The tumor microenvironment (TME) of GBM is a fundamental regulator of tumor progression and invasion and therapeutic efficacy. It is composed of several stromal cell types, including astrocytes, macrophages, pericytes, fibroblasts, and endothelial cells 7, 8. In GBM, up to one-half of the cells of the tumor mass are tumor-associated macrophages and microglia (TAMs), which exert a tumor-supportive role in GBM progression and inversely correlate with GBM prognosis 9, 10. TAMs are functionally plastic and switch the polarization status between the classically activated M1 phenotype and alternatively activated M2 phenotype, exerting antitumorigenic and protumorigenic activities, respectively 11. Microglia and macrophages are the main immune infiltrating cells in the TME of GBM, and both microglia and macrophages can be polarized into anti-tumor M1 type or tumor-promoting M2 type by different stimulations 12, 13. Therefore, targeted modulation of microglia and macrophages to rebuild the immune response balance is a promising antitumor strategy for GBM to improve outcomes 7, 14. Current microglia/macrophage-based immunotherapy strategies mainly include depletion of microglia and macrophages in the TME and regulation of the cell phenotype to the treatment of tumors with poor immunogenicity 13, 15.

In recent years, cancer immunotherapy based on immune checkpoint blockers (ICBs) has garnered considerable attention, and the immune normalization approach has demonstrated significant antitumor activity in a variety of tumors 16. Systemic administration of anti-cytotoxic T lymphocyte-associated protein 4 (anti-CTLA-4) or anti-programmed death-1 (PD-1)/programmed death ligand 1 (PD-L1) monoclonal antibodies (mAbs) can suppress some tumors. However, clinical trials focusing on brain tumors have yielded mediocre results partly because gliomas are considered “cold” predominant accumulation of immunosuppressive TAMs in GBM and poorly infiltrated with lymphocytes 10, 17. In addition to targeting adaptive immune checkpoints, recent studies have established the usefulness of targeting phagocytosis checkpoints, which stimulate both the innate and adaptive immune systems to generate antitumor responses and prove beneficial to patients with cancer 18-20.

Phagocytosis is a complex process that requires both the activation of pro-phagocytic “eat me” signals and the simultaneous disruption of anti-phagocytic “don't eat me” signals 21. Normal healthy cells except aging red blood cells lack “eat me” signals, whereas all human solid tumor cells express the “don't eat me” signal transmembrane protein CD47 to evade phagocytosis, providing a potential immunotherapeutic strategy for cancer treatment without unacceptable toxicity 22. The CD47-signal regulatory protein alpha (SIRPα) pathway is one of the most studied phagocytosis checkpoints in macrophages. Macrophage-mediated phagocytosis of tumor cells via blockade of the anti-phagocytic CD47-SIRP interaction using anti-CD47 antibodies has shown potential in preclinical xenografts of various human malignancies 23, 24 Moreover, traversing the BBB for mAb-mediated treatment of GBM is another challenge that largely precludes the systemic approach for mAb therapy in most patients with GBM 25. Glioma cells induce the expression of the surface protein CD47 (the “don't eat me” signal), which inhibits TAM phagocytic activity 22, 26. Furthermore, anti-CD47 antibody therapy has been demonstrated to bridge the innate and adaptive responses and requires the expression of the cytosolic DNA sensor stimulator of interferon genes (STING) 27. In recent years, STING has emerged as a promising target for cancer immunotherapy which triggers the secretion of type I interferon (IFN-I) and pro-inflammatory cytokines to elicit antitumor immune responses 28. STING agonists, such as cyclic dinucleotides (CDNs) due to their low bioavailability, high toxicity, and poor cellular targeting hinder their further clinical application 29. Administration of a STING agonist (cyclic-di-GMP; CDG) can support innate immune function in GBM and improve the survival of glioma-bearing mice 30. However, the existence of BBB has long been a challenge for systemically delivering drugs, including larger molecules such as immune checkpoint blockade antibodies, which fail to pass the BBB, thereby preventing effective treatment of brain-related diseases 31. The rapid development of nanotechnology has shed new light on therapeutic strategies for drug delivery to cancer. A wide variety of nanocarriers have been designed to improve the efficiency and accuracy of drug delivery, including polymeric nanoparticles, nanocapsules (NAcp), lipid nanoparticles, metallic nanoparticles, micelles, and dendrimers 32-34. Novel and highly specific nanocarriers with reduced systemic toxicity and the ability to bypass BBB have been implicated in targeted drug delivery for GBM treatment 13, 24, 35-40. It has been reported that acetylcholine and choline analogues can interact with nicotinic acetylcholine receptors (nAChRs) and choline transporters to effectively penetrate the BBB by in situ polymerization-formed nanocapsules that encapsulate macromolecules, proteins, RNA, or DNA and protect them from the physiological environment, and deliver therapeutics to the central nervous system (CNS) 41, 42. Nevertheless, the relatively low efficiency, in vivo instability, and degradation of cargo of various carrier vehicles may hamper the therapeutic outcome, thus requiring further improvements in the design of nanodelivery strategies 43.

**Scheme 1:**
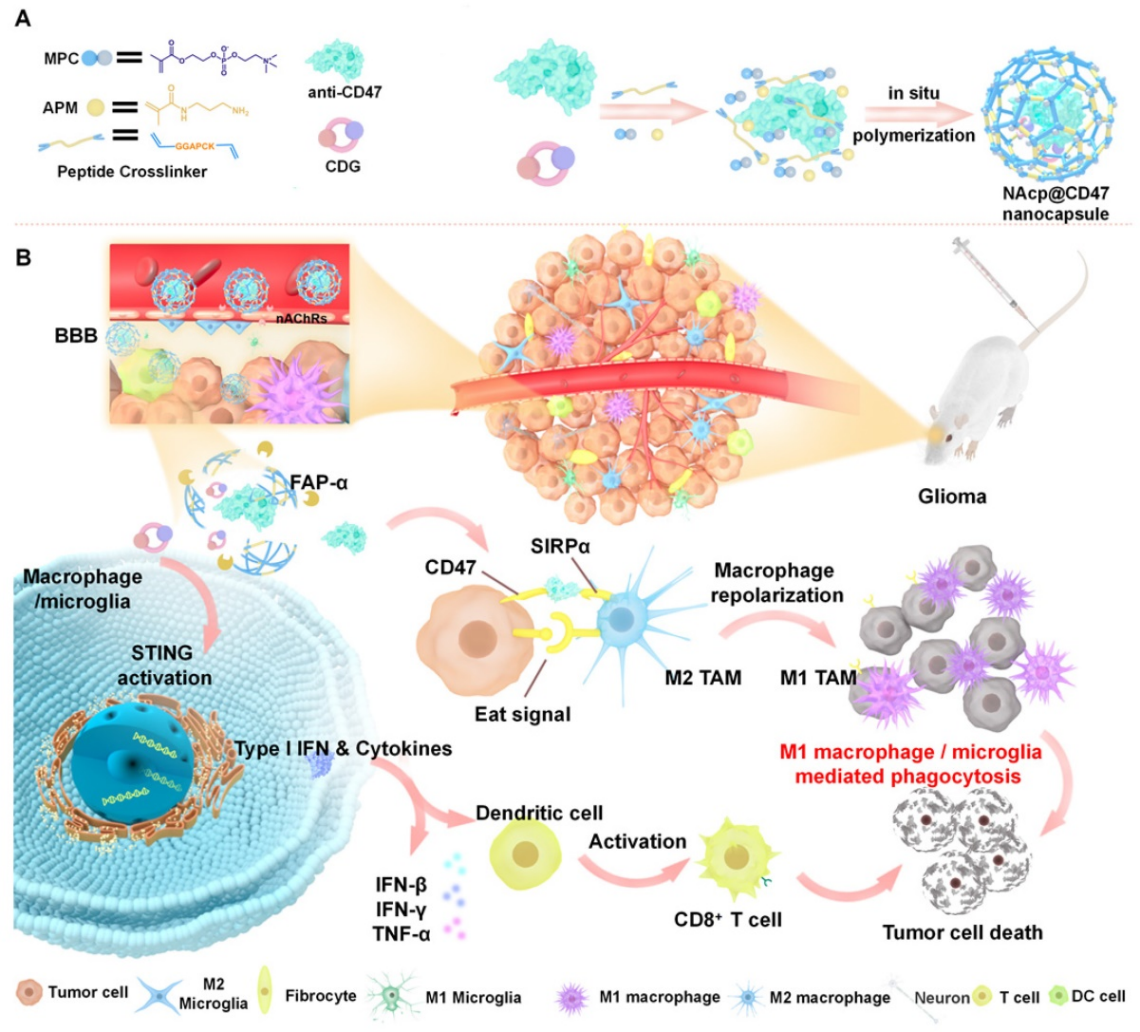
Schematic illustration of the NAcp@CD47 nanocapsule for dual delivery of anti-CD47 antibodies and STING agonists for GBM immunotherapy by repolarization of macrophages and promotion of phagocytosis. **(A)** The process of constructing the FAPá responsive prodrug nanocapsule: anti-CD47 antibodies and CDG are co-encapsulated in NAcp@CD47 using MPC, APM and FAP-α-responsive peptide crosslinker in situ polymerization nanocapsule. **(B)** The mechanism of NAcp@CD47 polarization of microglia and macrophages against GBM. After intravenous injection, NAcp@CD47 can be delivered to the CNS crossing the BBB mediated by MPC, release anti-CD47 antibodies and CDG after FAP-α enzymatic degradation in the GBM microenvironment to block the phagocytosis checkpoint CD47-SIRPα and promote the production of IFNs via the STING signaling pathway. Consequently, the reprogrammed microglia (or macrophages) enhances the phagocytosis of cancer cells. Similarly, IFNs facilitate increased infiltration of immune cells and increase immunogenicity to convert “cold” tumors into “hot” tumors.

Herein, we prepared and characterized a novel biomimetic nanocapsule “NAcp@CD47”, which forms a thin polymer shell that can effectively penetrate the BBB and deliver encapsulated anti-CD47 antibodies and STING agonists in GBM to activate phagocytosis (Scheme 1). The shell is formed using in situ polymerization of monomers and stabilized by tumor environmentally responsive crosslinkers and cargoes can be trigger-released only by the fibroblast activation protein α (FAP-α) cleavage of these crosslinkers. The released anti-CD47 antibodies reprogram microglia and macrophages from M2 cells toward an M1-like phenotype to enhance the phagocytosis of GBM tumors. Furthermore, CDG stimulated the production of IFNs via the STING pathway, which synergistically re-educates TAMs to a pro-inflammatory phenotype, as well as synergizes M1 microglia and macrophages to phagocytosis of tumor cells in the orthotopic GBM models to substantially improve anti-CD47 blockade efficacy.

## Methods

### Materials, cell lines and animals

All chemicals, if not specified, were purchased from Sigma-Aldrich and used without purification, including immunoglobulin G (IgG) from mouse serum, ammonium persulfate (APS), N,N,N',N'-tetramethylethylenediamine (TMEDA), Cyclic-di-GMP (CDG), 3% thioglycollate broth, papain, and Cell Counting Kit-8 (CCK-8). Fmoc-amino acids were purchased from GL Biochem (Shanghai) Ltd. N,N-dimethylformamide (DMF) and dichloromethane (DCM) were purchased from Tianjin Damao Chemical Reagent Factory. Piperidine was purchased from Shanghai Sinopharm Chemical Reagent Co., Ltd. Trifluoroacetic acid (TFA) and CellTracker™ Blue (7-amino-4-chloromethylcoumarin) (CMAC, cat. no. C2110) were purchased from Thermo Fisher Scientific. Anti-CD47 antibody (mAb: MIAP301) was purchased from Biolegend (cat. no. 127518, Clone, miap301). Anti-CD47 antibody-APC (aCD47-APC) (cat. no. 17-0479-42, Clone, B6H12) was purchased from Invitrogen. N-(3-aminopropyl) methacrylamide (APM) was purchased from Beijing Innochem Science & Technology Co., Ltd. 2-methacryloyloxyethyl phosphorylcholine (MPC) was purchased from Shanghai Yuanye Bio-Technology Co., Ltd. 1,1-dioctadecyl-3,3,3,3-tetramethylindotricarbocyanine iodide (DiR) was purchased from AAT Bioquest Inc. FAPα enzyme was purchased from ACROBiosystems Inc. The transwell with permeable polyester membrane inserts was purchased from Corning. GFP/LUC lentivirus was purchased from GenePharma (Shanghai, China). Mouse TNF-α ELISA Kit and mouse IFN-γ ELISA Kit were purchased from Beyotime Biotechnology (Shanghai, China). Mouse IFN-β ELISA kit was purchased from Elabscience (cat. No. E-EL-M0033c). In Situ Cell Death Detection Kits were purchased from Merck. CD206 (cat. no. 141716, Clone, C068C2), CD80 (cat. no. 105027, Clone, GL-1), F4/80 (cat. no. 123116, Clone, BM8), CD11b (Biolegend, cat. no. 101208, Clone, M1/70), CD45 (Biolegend, cat. no. 103108, clone 30-F11), CD3 (cat. no. 100204, Clone, 17A2), and CD8 (cat. no. 140408, Clone, 53-5.8) were purchased from Biolegend. CD47 (cat. no. SC-53050, Clone, 16423) was purchased from Santa Cruz Biotechnology, Inc. iNOS [RM1017] (ab283655), ARG1 [EPR6671(B)] (ab124917), IBA1 [EPR16589] (ab283319), CD8 [EPR22331-81] (ab263946) and Anti-Nicotinic Acetylcholine Receptor alpha 5/CHRNA5 [EPR24135-98] (ab259859) were purchased from Abcam. α-Tubulin (AF0001) and GAPDH (AF0006) were purchased from Beyotime Biotechnology (Shanghai, China). Hyoscyamine (Daturine), S4014, selleck, China. Fibroblast activation protein-α (FAP) Rabbit pAb (A6349), ABclonal, Inc., China.

Mouse glioblastoma cell line GL261 was purchased from Cell Bank of Chinese Academy of Sciences. bEnd.3 cells were purchased from Jiangsu KeyGEN BioTECH Co., Ltd. Cells were cultured in Dulbecco's Modified Eagle Medium (DMEM) (Gibco) supplemented with 10% (v/v) fetal bovine serum (FBS) (Gibco), 100 U/mL penicillin (Gibco) and 100 μg/mL streptomycin (Gibco) at 37℃ under 5% CO_2_ in a humidified incubator. 0.25% (w/v) trypsin solution was purchased from Gibco. Male BALB/c and BALB/c nude mice (6-10 weeks) were purchased from Charles River Laboratories (Beijing, China). eGFP transgenic mice were purchased from Cyagen Biosciences (Guangzhou, China). All mouse studies were carried out following the protocols approved by the Institutional Animal Care and Use Committee at the National Center for Nanoscience and Technology of China (approval number: NCNST21-2110-0604). Primary mouse astrocytes were obtained from the brains of 1-day-old in BALB/c mice as previously reported 44 with some modification. Macrophages were harvested from peritoneal cavity by wash after injection of 3% thioglycollate broth. GL261 cells infected with lentivirus were sorted using FACSAria III (Becton Dickinson).

### Preparation of nanocapsule

Anti-CD47 antibody (aCD47) or anti-CD47 antibody-APC (aCD47-APC) was dissolved in PBS (20×10^-3^ M, pH 7.4) with a concentration of 2.03 mg/mL. APM was first added (aCD47 or aCD47-APC: APM, 1:500, n/n) and stirred for 10 min at room temperature. Subsequently, the main monomer MPC, FAPα-responsive peptide crosslinker and CDG were added (MPC: APM: crosslinker, 10:1:1, n/n) (aCD47: CDG, 5:1, w/w) under stirring. Finally, the initiating system APS and TEMED were added (APS:aCD47, 1:000:1, n/n) (APS:TEMED, 2:2, w/w) to initiate the polymerization. The final concentration of aCD47 or aCD47-APC was controlled to be 1 mg/mL and the polymerization time was about 24 h at 4˚C. Synthesized nanocapsules were dialyzed against PBS and purified by passing through a hydrophobic interaction column (Phenyl-Sepharose 4BCL). Since the nanocapsules possess a super-hydrophilic surface, their binding affinity to the column is much weaker than the native mAb. NAcp@CD47 and control group for in vivo studies were both encapsuled with near-infrared dye DiR. DiR needed to be ultrasonically mixed with MPC first for 10 min, and then be added to the system together with MPC. The nanocapsules were analyzed using dynamic light scattering (DLS) to measure size and zeta potential, and transmission electron microscopy (TEM) to analyse particle morphology. The CDG loading efficiency (>80%) was quantified using high-performance liquid chromatography.

### Preparation of liposome

2 mg of DOPE was resolved in 1 mL of trichloromethane, 2.34 mg of DSPE-PEG2000 was resolved in 1 mL of trichloromethane and 1.03 mg of cholesterol was resolved in 1 mL of trichloromethane. The mix was evaporated in rotary evaporator. Same amout of aCD47-APC was resolved into 2 mL of PBS, shaken in water bath under 45℃ for 30 min before 10 min of ultrasound. 0.22 μm of filter was used for several times, and TEM was used to analyze particle morphology.

### BBB permeability in vitro

The mouse brain endothelial bEnd.3 cells were employed to establish an in vitro BBB model as previously reported 45. bEnd.3 cells were cultured on the transwell membrane (pore diameter of 0.4 μm) above GL261 GBM cells. When reached confluence, the cells formed a monolayer, which was reasonably regarded as the BBB in vitro. The cells were used for further experiments when the transendothelial electrical resistance was at least 200 Ω•cm^2^. To evaluate NAcp@CD47 permeation across this monolayer, we synthesized and used aCD47 labeled with APC (aCD47-APC) instead of aCD47 to encapsule NAcp@CD47-APC. The bottom chamber was imaged under a Leica TCS SP8 confocal microscope. The recorded images were analyzed with ImageJ software.

### BBB permeability in vivo

DiR labeled NAcp@CD47 and DiR/PBS (equivalent amounts of DiR dye) were injected into male BALB/c nude mice (6-10 weeks) through the tail vein (n = 3 for each group). Brain fluorescence images were acquired 1, 2, 4, 8, 12, 24, 36 and 48 h after injection using an in vivo imaging system (IVIS) (Perkin Elmer). In the meantime, 10 μL of blood samples from the tail vein at different time points were collected and diluted in PBS, and the release rate of NAcp@CD47 in vivo was recorded with the IVIS. As GL261 cells expressed luciferase, related substrates were also administrated intravenously into the mice for the orientation of brain glioma. Furthermore, samples in the brain, heart, liver, spleen, lung and kidney were obtained for ex vivo imaging 48 h after administration. The recorded images were analyzed with Living Image @ 4.3.1 software.

### Cytotoxicity in vitro

The cytotoxicity of NAcp@CD47 toward GBM and normal cells was assessed by CCK-8 assay. GL261 GBM cells or primary mouse astrocytes were seeded in a 96-well plate at a density of 5000 cells per well and incubated for 24 h. Then the culture medium was removed and 100 μL of cell culture medium containing various concentrations of NAcp@CD47 (0-25 μg/mL) was added to each well. After 24 or 48 h of incubation at 37℃, 10 μL of CCK-8 was added to each well. After another 1.5 h of incubation, the 96-well plates were placed in a microplate reader (FlexStation 3, Molecular Devices) to measure the absorbance at 450 nm.

### Release of NAcp@CD47 in vitro

To verify the FAP-α-responsive dissociation behavior of the nanocapsules, the release of NAcp@CD47 was studied at 37℃ in ACSF or PBS (25 μg/mL) containing FBS with or without FAP-α enzyme. The release profiles of aCD47 and CDG were quantified using high performance liquid chromatography (HPLC) (Alliance HPLC systems, Waters Co.) using a C-18 column at 260 nm. Water and acetonitrile were used as mobile phases. The results were quantified using a standard curve of a serial dilution of stock solutions and normalized to total cumulative release.

### In vitro phagocytosis assay

Peritoneal macrophages and brain microglia were isolated and prepared from eGFP transgenic mice as previously described 46. GL261 GBM cells were stained with Hoechst 33342. Then cancer cells (4×10^5^) were co-cultured with cultured peritoneal macrophages or brain microglia (1×10^5^) in serum-free medium containing NAcp@IgG or NAcp@CD47 and enzymes. After incubation for 30 min at 37℃, phagocytosis was studied by fluorescence microscopy (THUNDER Imager Tissue, Leica) and flow cytometry (FACSCantoTM II (Becton Dickinson).

### In vivo tumor models and treatment

To study the therapeutic effects of NAcp@CD47 on tumor growth, 18 male BALB/c mice (6-10 weeks) BALB/c mice housed under specific pathogen-free (SPF) conditions were used to establish a tumor-bearing mouse model. GL261 GBM cells (1×10^5^ cells suspended in 200 μL PBS) were engrafted subcutaneously into each mouse. All animal protocols were approved by the Institutional Animal Care and Use Committee of the National Center for Nanoscience and Technology (IACUC Issue No. NCNST21-2110-0604). On day 7, mice were divided into 3 groups (n = 6) randomly. From day 10, mice were intravenously injected with 100 μL NAcp@CD47 (5 mg/kg) or PBS or aCD47 control (25 µg) (n = 6 mice in each group) through the tail vein twice per week. After total 7 times of treatments had finished, mice were sacrificed and tumors were dissected, measured, and weighted. The tumor size was measured and calculated according to the following formula: width^2^ × length × 0.5.

### Cytokine detection

The blood samples from the mice of each group were collected by retro-orbital sinus puncture. The serum levels of IFN-γ (Beyotime Biotechnology, cat. No. PI508) and TNF-α (Beyotime Biotechnology, cat. No. PT512) were measured with commercial ELISA kits according to the manufacturer's instructions. 2×10^6^ macrophages (RAW 264.7 cells) and microglia (BV2 cells) were seeded in a 96-well plate, and then different concentrations of NAcp@CD47 or controls were co-incubated with the cells for 24 h. IFN-β was measured by mouse ELISA kit (Elabscience, cat. No. E-EL-M0033c).

### Western blot analysis

Total proteins were harvested and lysed by RIPA buffer (150 mM NaCl, 0.5% EDTA, 50 mM Tris, 0.5% NP40). The protein samples were loaded and separated by using SDS-polyacrylamide gel electrophoresis (PAGE). The proteins were then transferred onto PVDF membrane, blocked with 5% non-fat milk for 2 h at room temperature, incubated with the indicated antibodies at 4℃ overnight, incubated with horseradish peroxidize-conjugated secondary antibodies for 1 h at room temperature, and determined by using an ECL chemiluminescence system.

### Flow cytometry

Tumors obtained from mice were divided into small pieces and homogenized in cold staining buffer to form single cell suspensions in the presence of digestive enzyme. Cells were stained with fluorescence-labelled antibodies following the manufacturer's instructions. The stained cells were measured using FACSCanto^TM^ II (Becton Dickinson) or FACSAria III (Becton Dickinson) and analyzed by the FlowJo software (version 10.0.7, TreeStar).

### Histopathology examination

The excised tumors were collected and fixed in 10% formalin and embedded in paraffin. Then 5 μm sections were prepared and placed on glass slides. After deparaffinized in xylen and dehydrated in graded alcohols, the slides were stained with hematoxylin and eosin (H&E) and observed under an optical microscope.

### Immunofluorescence staining

Tumors were harvested from the mice in different groups, fixed with 4% paraformaldehyde for at least 24 h, dehydrated through gradient sucrose solution, and frozen in optimal cutting temperature (OCT) medium. Tumors were cut via a cryotome, mounted on slides and stained with primary antibody overnight at 4℃. After washing three times in TBST, secondary antibodies were incubated for 1 h at room temperature. Images were acquired on fluorescence microscopy (THUNDER Imager Tissue, Leica).

### TdT-mediated biotin-dUTP nick-end labelling (TUNEL) assay

Apoptosis cells in samples were examined with the TUNEL assay (Beyotime Biotechnology, cat. No. C1086; Roche, cat. No. 12156792910) according to the manufacturer's instructions and detected under fluorescence microscopy (THUNDER Imager Tissue, Leica).

### Orthotopic syngeneic model for brain tumors

GL261 cells transduced with the lentivirus stably expressing GFP and luciferase (GL261-GFP/LUC) were orthotopically inoculated into 6- to 8-week-old BALB/c mice to establish a brain tumor model. Briefly, mice were anesthetized using isoflurane and placed in a stereotactic frame. A burr hole was drilled through the skull at a position 2 mm lateral and 2 mm posterior of bregma. Stereotaxic injection of GL261-GFP/LUC cells (1×10^4^ in 10 μL total volume/mouse) was performed at a depth of 3 mm. 14 days later, mice were intravenously injected with 100 μL NAcp@CD47 or PBS or aCD47 control through the tail vein twice per week for a total of 7 times. The tumor was observed using an in vivo imaging system (IVIS) (Perkin Elmer). 10 minutes after intraperitoneal injection of D-luciferin (Thermo Scientific Pierce) in DPBS (15 mg/mL) into each mouse at a dose of 10 μL/g, mice were imaged with 1 minute of exposure time.

### Statistical analysis

All statistical analyses were performed using GraphPad Prism 7 software. Data were presented as mean ± SD. Significance comparisons between groups were calculated by one-tailed unpaired t-test with Welch's correction. Survival analysis was performed using a log-rank test. P < 0.05 was considered significant if not stated otherwise.

## Results and Discussion

### Preparation and characterization of NAcp@CD47

The nanocapsules were prepared using a simple encapsulation technique 47, 48, as illustrated in Scheme 1A. To enhance cancer immunotherapy responses, a combination of anti-CD47 antibodies and STING activator CDG (cyclic di-GMP) was encapsulated into the nanocapsules 49. N-(3-aminopropyl) methacrylamide (APM), 2-methacryloyloxyethyl phosphorylcholine (MPC), and protease-degradable crosslinkers via electrostatic and hydrogen-bonding interactions enrich the monomers and crosslinkers around the protein molecules. In situ free-radical polymerization formed a thin polymer layer around the anti-mouse CD47 antibodies and STING activator cyclic diguanylate monophosphate (cyclic-di-GMP or CDG), forming the nanocapsules termed NAcp@CD47. MPC, a zwitterionic polymer containing a choline and acetylcholine analogue through interaction with nicotinic acetylcholine receptors and choline transporters to bypass BBB, was employed because of its superior BBB penetration and biocompatibility properties 39, 50. To ensure specific controlled drug release, we designed a FAP-α substrate responsive linker, which is highly expressed on the surface of cancer-associated fibroblasts (CAFs) in most cancers including GBM, but is scarcely expressed by normal tissues 51-53. We synthesized and modified a unique peptide specifically cleaved as targeted by the FAP-α enzyme. The biacrylated FAP-α-responsive peptide crosslinker (azidoacetic acid-GGAPCK-azidoacetic acid) was synthesized using solid phase and characterized using electrospray ionization mass spectrometry (ESI-MS) (Figure S1).

**Figure 1:**
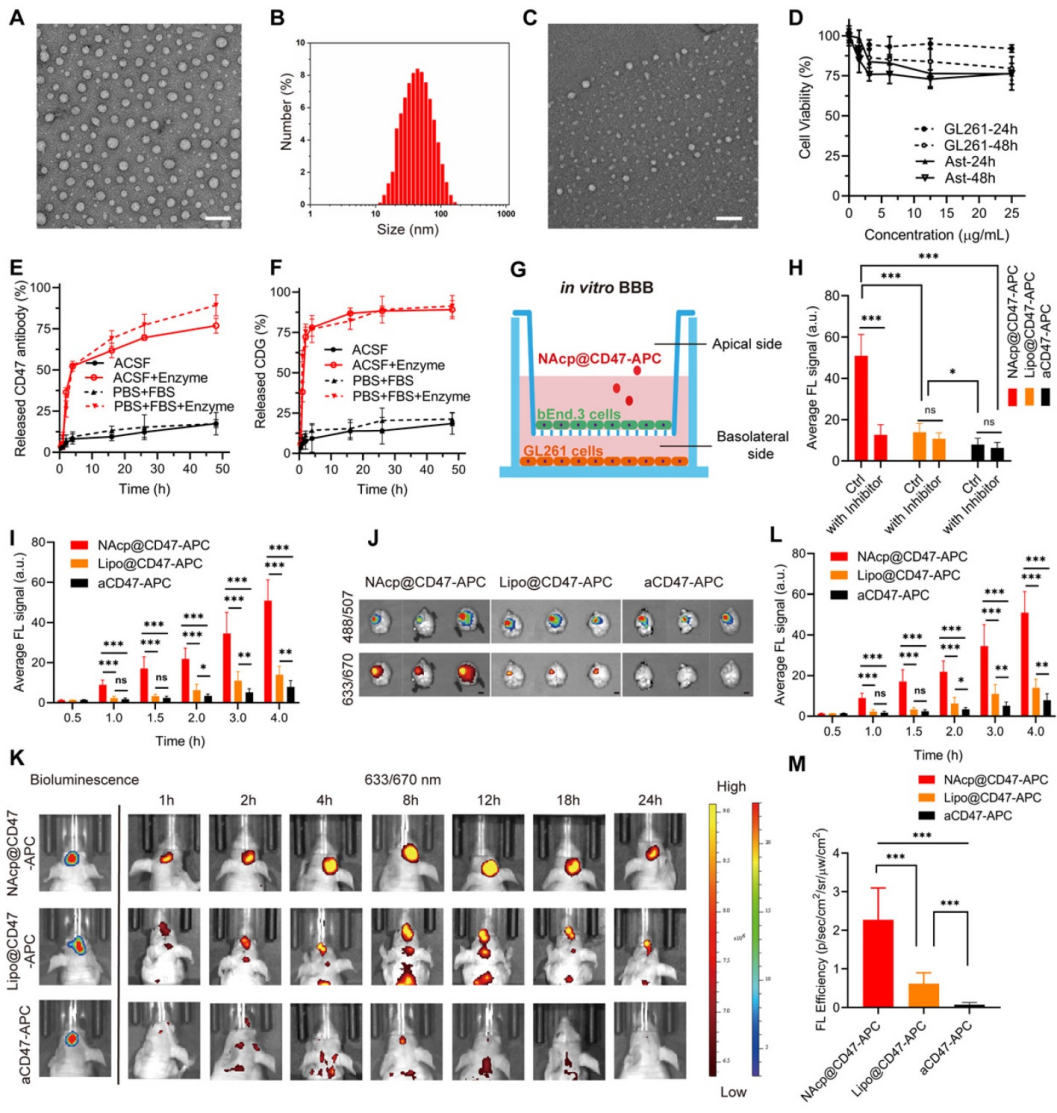
Characterization of NAcp@CD47. **(A)** TEM images of NAcp@CD47 (scale bar, 200 nm). **(B)** The average hydrodynamic size of NAcp@CD47 determined using dynamic light scattering (DLS). **(C)** TEM images of NAcp@CD47 after FAP-α enzymatic degradation. **(D)** Cell viability of GL261 glioblastoma cells and primary mouse astrocytes after incubation with various concentrations of NAcp for 24 or 48 h (n = 3, mean ± SD). **(E-F)** Cumulative release profiles of aCD47 (E) or CDG (F) from the NAcp@CD47 dispersed in artificial cerebrospinal fluid (ACSF) or PBS (25 ìg/mL) containing FBS with or without FAP-α enzyme at 37℃ as determined by high performance liquid chromatography (HPLC). **(G)** Schematic of the in vitro BBB model. **(H)** Average fluorescence signal of GL261 GBM cells in the in vitro BBB model after 4 h of incubation with NAcp@CD47-APC, Lipo@CD47-APC, or aCD47-APC with or without nAChR inhibitor. **(I)** Average fluorescence signal of GL261 GBM cells in the in vitro BBB model after 0 to 4 h of incubation with NAcp@CD47-APC, Lipo@CD47-APC, or aCD47-APC. **(J)** Ex vivo fluorescence imaging of brains from mice after injection of NAcp@CD47-APC, Lipo@CD47-APC, or aCD47-APC. Excitation/emission wavelengths 488/507 nm for GFP and excitation/emission wavelengths 633/670 nm for APC revealed a colocalization of GBM and NAcp@CD47 (scale bar, 2 mm). **(K)** In vivo fluorescence imaging of mice at indicated time points after intravenously injected with NAcp@CD47-APC, Lipo@CD47-APC, or aCD47-APC. **(L-M)** Quantitative analysis of in vivo (L) and ex vivo (M) fluorescence efficiency of NAcp@CD47 and control groups in the brain. *P < 0.05; **P < 0.01; ***P < 0.001; ns, not significant.

Transmission electron microscopy (TEM) observation and dynamic light scattering (DLS) measurements showed that monodisperse NAcp@CD47 were spherical and uniformly distributed with a hydrodynamic diameter of 50.7 nm (Figure 1A-B). Nanocapsules without cargoes displayed a similar round morphology and diameter (Figure S2). The successful encapsulation of NAcp@CD47 was also identified by changes in zeta potential (Figure S3) and Coomassie brilliant blue (CBB) staining of CD47 using SDS-polyacrylamide gel electrophoresis (PAGE) (Figure S4). Environmental responsive carriers often enable more precise and controlled drug release at the targeted site. As illustrated in Figure 1C, the morphology of NAcp@CD47 was constantly reduced and degraded after the addition of enzymes. Thus, antibody drugs were gradually released from the nanocapsules with the assistance of tumor microenvironment overexpressing FAP-α enzymes. After incubation with various concentrations of NAcp@CD47 for 24 or 48 h, extremely low cytotoxicity was observed in both GL261 GBM cells and primary mouse astrocytes (Figure 1D). In the presence of the FAP-α enzyme, CD47 (Figure 1E) and CDG (Figure 1F) were gradually released from the nanocapsules in both artificial cerebrospinal fluid (ACSF) and phosphate-buffered saline (PBS) containing fetal bovine serum (FBS), which were used to imitate brain and blood environment respectively, whereas a minimal of cargoes were released in both solutions without enzyme. This work demonstrates the feasibility of encapsulating proteins within the nanocapsules and releasing their protein cargo with a controlled release rate following enzymatic degradation of the nanocapsules.

The ability of NAcp@CD47 to cross the BBB-mimicking cell monolayer was evaluated in an in vitro BBB model (Figure 1G). The expression of nAChRs was observed on mouse brain capillary endothelial bEnd.3 cells in this model (Figure S5). CD47 antibodies labeled with allophycocyanin (APC) together with CDG encapsulated as NAcp@CD47-APC were added to the supernatant and fluorescence images of GL261 cells in the bottom chamber at different time points were measured (Figure S6). Similar amounts of APC-labeled CD47 antibodies together with CDG encapsulated into liposomes (Lipo@CD47) or in free state (aCD47) were used as controls. Compared to the control groups, the BBB penetration of NAcp@CD47 dramatically increased with incubation and demonstrated excellent permeability (Figure 1H), indicating the importance of the presence of MPC on the surface. Treatment of nAChR inhibitor Hyoscyamine (Daturine) significantly reduced the BBB permeability of NAcp@CD47, suggesting the roles of MPC-mediated targeting delivery crossing the BBB. The cargoes were also encapsulated into liposomes as controls (Figure S7), and the results showed that the BBB penetration was not affected by the nAChR inhibitor. The ablity of Lipo@CD47 group to penetrate the BBB was higher than that of aCD47 group but extremely lower than that of NAcp@CD47 group. Accumulative effects of BBB penetration were observed in all three groups (Figure 1I). The ability of NAcp@CD47 to cross the BBB in vivo was further assessed. To better evaluate the penetration efficiency, GL261 cells expressing luciferase (LUC) and eGFP were contructed so that the location of GBM could be observed. Consistent with the in vitro results, substantial fluorescence was observed in the brains of mice injected with NAcp@CD47 (Figure 1K). The higher brain targetability of NAcp@CD47 was also confirmed using ex vivo imaging of brains collected from mice sacrificed 48 h post-injection (Figure 1J). Quantitative analysis showed that the fluorescence efficiency of NAcp@CD47 in the brain was higher than that in the control (Figure 1L-M), indicating that the specific recognition between MPC in NAcp@CD47 and nAchR on the cell surface greatly facilitated BBB penetration. We also investigated the in vivo BBB penetration of NAcp@CD47 and the control group both encapsulated with the near-infrared dye 1,1-dioctadecyl-3,3,3,3-tetramethylindotricarbo-cyanine iodide (DiR), a molecule with stronger in vivo tracking ability (Figure S8). The targeting effects of NAcp@CD47-APC, Lipo@CD47-APC, or aCD47-APC were also evaluated in subcutaneous implanted tumor model mice via tail vein injection (Figure S9). These results validate that NAcp@CD47 mimicking both structural and functional aspects of natural nicotinic acetylcholine exhibits superior BBB penetration and brain targeting.

### In vitro phagocytosis of GBM mediated by NAcp@CD47

**Figure 2:**
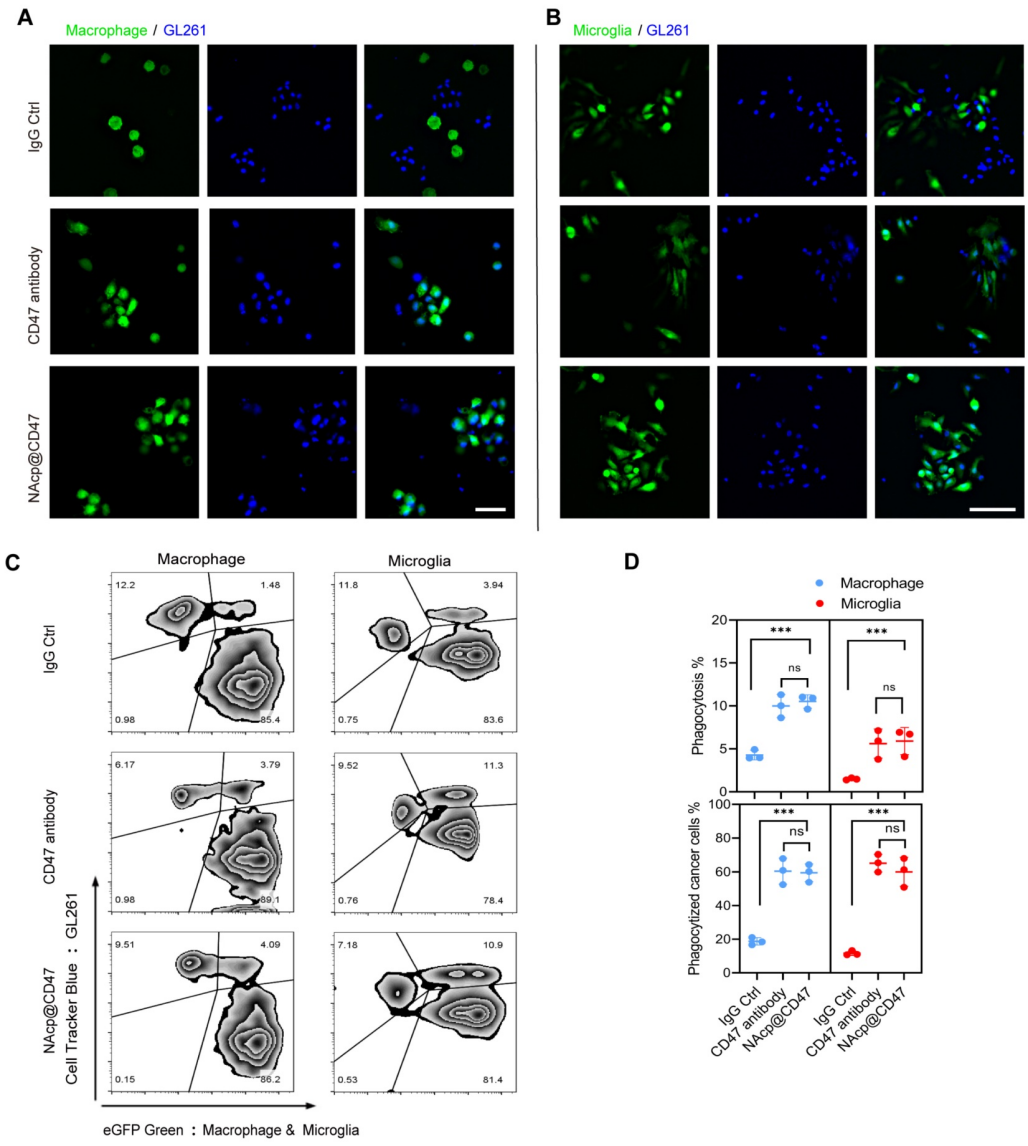
NAcp@CD47-mediated phagocytosis in vitro. **(A-B)** Representative fluorescence images of phagocytosis assays, in which peritoneal macrophage (A) and brain microglia (B) from eGFP transgenic mice displayed green were co-cultured with GL261 GBM cells labelled with Hoechst 33342 (blue) for 30 min in the presence of NAcp@CD47, CD47 antibody or IgG control. Scale bar, 200 ìm. Experiments were repeated three times. **(C-D)** Representative flow cytometric analysis images (C) and relative quantification (D) of the phagocytosis of GL261 GBM cells by peritoneal macrophage and brain microglia in the presence of NAcp@CD47, CD47 antibody or IgG control. Data are presented as mean ± SD (n = 3). Phagocytosis or phagocytized cancer cells were quantified as the percentage of double-positive cells. ***P < 0.001; ns, not significant.

To determine the ability of NAcp@CD47 to antagonize cell-surface CD47, we investigated whether NAcp@CD47 potentiates tumor cell phagocytosis in vitro. Peritoneal macrophages and brain microglia were isolated and cultured from eGFP transgenic mice (Figure S10), and co-cultured with GL261 GBM cells labelled with Hoechst 33342 or CellTracker^TM^ Blue, with the addition of FAP-α enzyme. Compared with isotype IgG antibody and CD47 antibody as controls, the presence of NAcp@CD47 increased the phagocytosis of GL261 GBM cells by macrophages (Figure 2A) and microglia (Figure 2B). Flow cytometry results also demonstrated that NAcp@CD47 enhanced the double positive signal (green and blue) of the mixtures (Figure 2C). Further quantitative analysis revealed that the percentages of phagocytosis and phagocytized cancer cells mediated by macrophages and microglia were significantly elevated in the presence of NAcp@CD47 (Figure 2D). Furthermore, a significant increase in the expression of IFN-β by NAcp@CD47 (Figure S11) indicated that NAcp@CD47 could enhance the delivery of CDG into DCs and TAM in vivo to induce the secretion of IFNs by STING activation. In contrast, anti-CD47 alone caused no significant changes in IFN-β. Thus, these results indicate that NAcp@CD47 can release CD47 antibodies and CDG, enhancing the phagocytosis of GBM cells by both macrophages and microglia in vitro.

### NAcp@CD47-mediated antitumor immune response in subcutaneously implanted tumors

**Figure 3:**
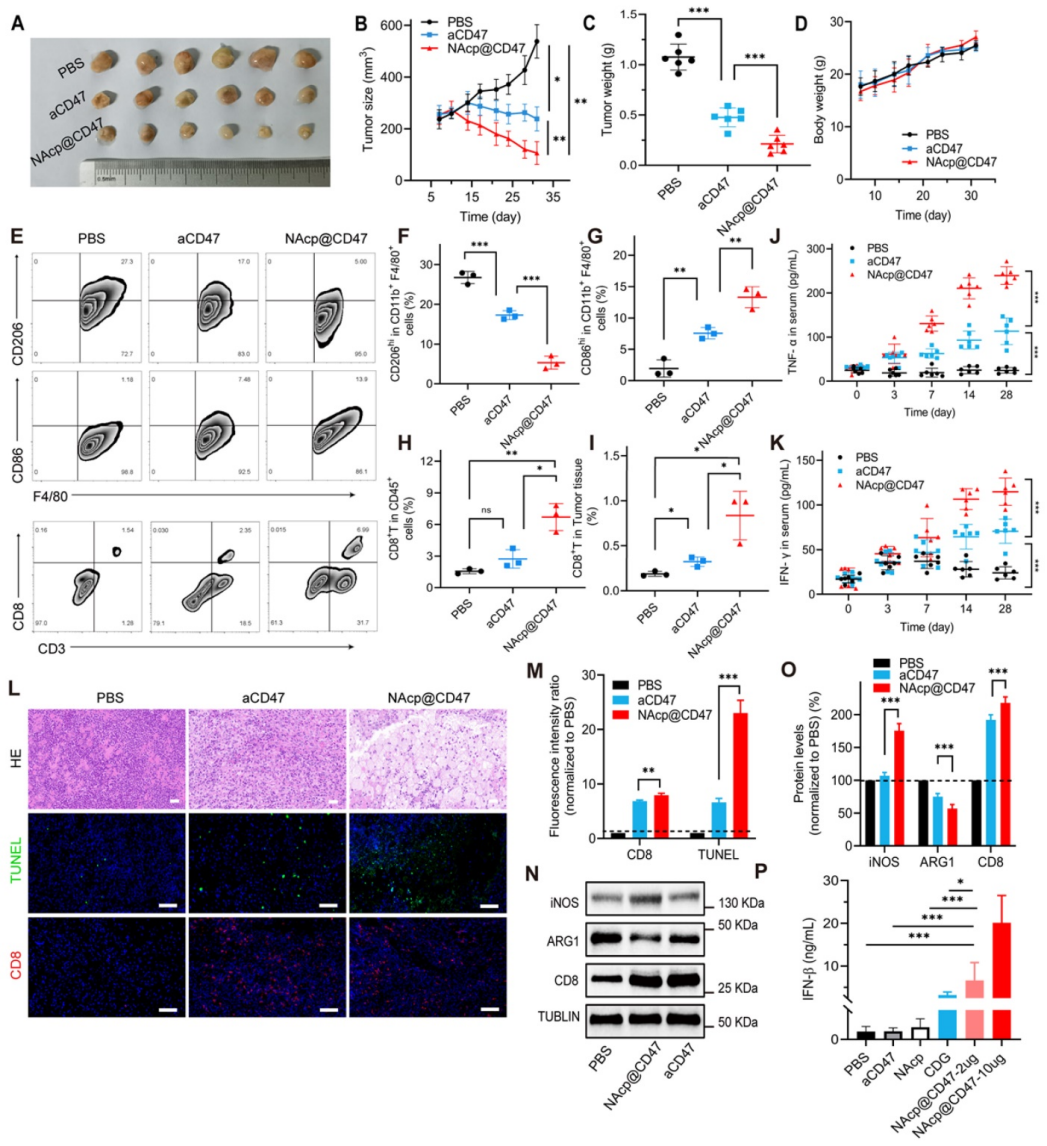
NAcp@CD47-mediated antitumor immune response in subcutaneously implanted tumors in vivo. **(A)** Photographic illustration of tumors harvested from mice intravenously injected with PBS, CD47 antibody control, and NAcp@CD47 at the end point. Each scale of the ruler represents 1 mm. **(B-C)** The tumor size (B) and tumor weight (C) of the excised tumors in different groups. **(D)** Weight changes of mice in distinct groups. Data are presented as mean ± SD (n = 6). **(E-I)** Representative flow cytometric analysis images (E) and percentages (F-I) of M2-like macrophages (CD206^hi^ in F4/80^+^CD11b^+^) and M1-like macrophages (CD86^hi^ in F4/80^+^CD11b^+^) and cytotoxic T lymphocytes (CD3^+^CD8^+^, CTLs). **(J-K)** The levels of tumor necrosis factor alpha (TNF-α) (J) and interferon-gamma (IFN-γ) (K) in the blood of mice before and after various treatments detected by ELISA (n = 6). Data are presented as mean ± SD (n = 6). **(L-M)** Representative H&E staining images and fluorescence microscopic images (L) of TUNEL assay and CD8^+^ cells in excised tumors of separate groups and relative quantification (M). **(N-O)** Relative quantification of the staining intensity (N) of the protein expression levels (O) of inducible nitric oxide synthase (iNOS), arginase-1 (ARG1), and CD8 in excised tumors of distinct groups analyzed by western blotting. **(P)** The levels of interferon-beta (IFN-β) in the tumor tissues. *P < 0.05; **P < 0.01; ***P < 0.001; ns, not significant.

Inspired by these findings that NAcp@CD47 could enhance tumor cell phagocytosis in vitro, we hypothesized that activation of macrophages mediated by the nanocapsules might exert antitumor activity in vivo. Notably, tumor growth was inhibited in tumor-bearing mice injected with NAcp@CD47 compared to mice injected with PBS or aCD47 controls (Figure 3A and Figure S12). The size and weight of the excised tumors were significantly lower in the NAcp@CD47-treated group than in the control groups (Figure 3B-C), whereas the body weights of mice in the different groups were not affected by the treatment (Figure 3D). Polarization of macrophages in tumors is important for cancer immunotherapy. To further investigate the immune effects mediated by NAcp@CD47 on subcutaneously implanted tumors, we examined the ability of NAcp@CD47 to modulate the polarization of macrophages in excised tumors. Because of the shared lineage of microglia and macrophages, the above markers are common to both cell types. We quantified the macrophage polarization inside the tumor by labeling the macrophages with CD86 (proinflammatory M1-like macrophages) and CD206 (anti-inflammatory M2-like macrophages) among the CD11b^+^F4/80^+^ cells. A significant reduction in immunosuppressive M2-like macrophages (CD206^hi^ in F4/80^+^CD11b^+^), and a significant increase in M1-like macrophages (CD86^hi^ in F4/80^+^CD11b^+^) were observed in the tumors harvested from mice treated with NAcp@CD47 as compared to controls (Figure 3E-I), which suggesting repolarization of macrophages with reduced immunosuppressive capacity and creating an innate inflammatory niche to prime adaptive immunity. Improving T cell infiltration within the TME is essential for effective cancer immunotherapy. Multiple studies have reported that activation of the STING pathway could reverse tumor immunosuppression to promote tumor lymphocyte infiltration (TILs) and elicit tumor-specific CD8^+^ T cells 54, 55. Flow cytometry analysis of tumors showed that NAcp@CD47 and aCD47 increased the relative ratio of infiltrating CD8^+^ T cell by about 4.53- and 2.97-fold compared with the untreated group and enhanced antitumor activity.

Blood samples from mice in different treatment groups were also collected to detect anti-tumor cytokines. The serum levels of IFN-γ and TNF-α were elevated gradually after NAcp@CD47 treatment and were significantly higher than those in the control groups (Figure 3J-K), which further confirmed the effective immunomodulatory capability of NAcp@CD47. The harvested samples were also stained with H&E for histological analysis. Enhanced anti-tumor activities and immune activation were observed in the group treated with NAcp@CD47 (Figure 3L). Immunofluorescence staining indicated that the number of TUNEL-positive cells and the percentage of CD8^+^ T cells were also increased in the tumors after NAcp@CD47 treatment as compared to controls (Figure 3M). Compared with the control group treated with PBS, obvious upregulation of inducible nitric oxide synthase (iNOS) and CD8 protein expression levels and downregulation of arginase-1 (ARG1) protein expression levels were observed in the excised tumors of NAcp@CD47-treated mice as analyzed using western blotting (Figure 3N-O). We observed that the M2-like TAMs (ARG1) were decreased and M1-like TAMs (iNOS) were increased in NAcp@CD47 group compared to aCD47 group. The findings indicated that more M1-type macrophages with anti-tumor effects were produced. In the NAcp@CD47 group, the levels of CD8^+^ T cells were increased and the immune activity was enhanced. Statistical analysis was performed and an extremely significant difference was found. The levels of IFN-β in tumor tissues were also detected by ELISA, and the data indicated that compared with controls NAcp@CD47 significantly increased the expression of IFN-β to efficiently activate the STING pathway (Figure 3P). Taken together, these results suggest that NAcp@CD47 induces activation of macrophages with an increased polarization of M1-phenotype TAMs and trigger a local immune response in the tumor microenvironment to inhibit the growth of subcutaneously implanted tumors, thus exerting therapeutic effects in vivo. Our study suggested that the combination of NAcp@CD47 is superior to soluble aCD47 in the reprogramming of microglia and macrophages from M2 cells toward an M1-like phenotype to enhance the therapeutic effect of cytotoxic T cells 56. All the above results suggest that NAcp@CD47 can remodel TAMs toward M1 phenotype, and further investigation was conducted in orthotopic syngeneic models of GBM.

### CD47 NAcp-mediated therapeutic effects in in vivo orthotopic syngeneic models for GBM

To further evaluate whether NAcp@CD47 could be effectively transported through the BBB and deliver the therapeutics to the central nervous system (CNS) for modulation phagocytosis, we established orthotopic syngeneic models with stereotaxic injection of GFP- and luciferase-labeled GL261 GBM cells into the brains of BALB/c mice.

**Figure 4:**
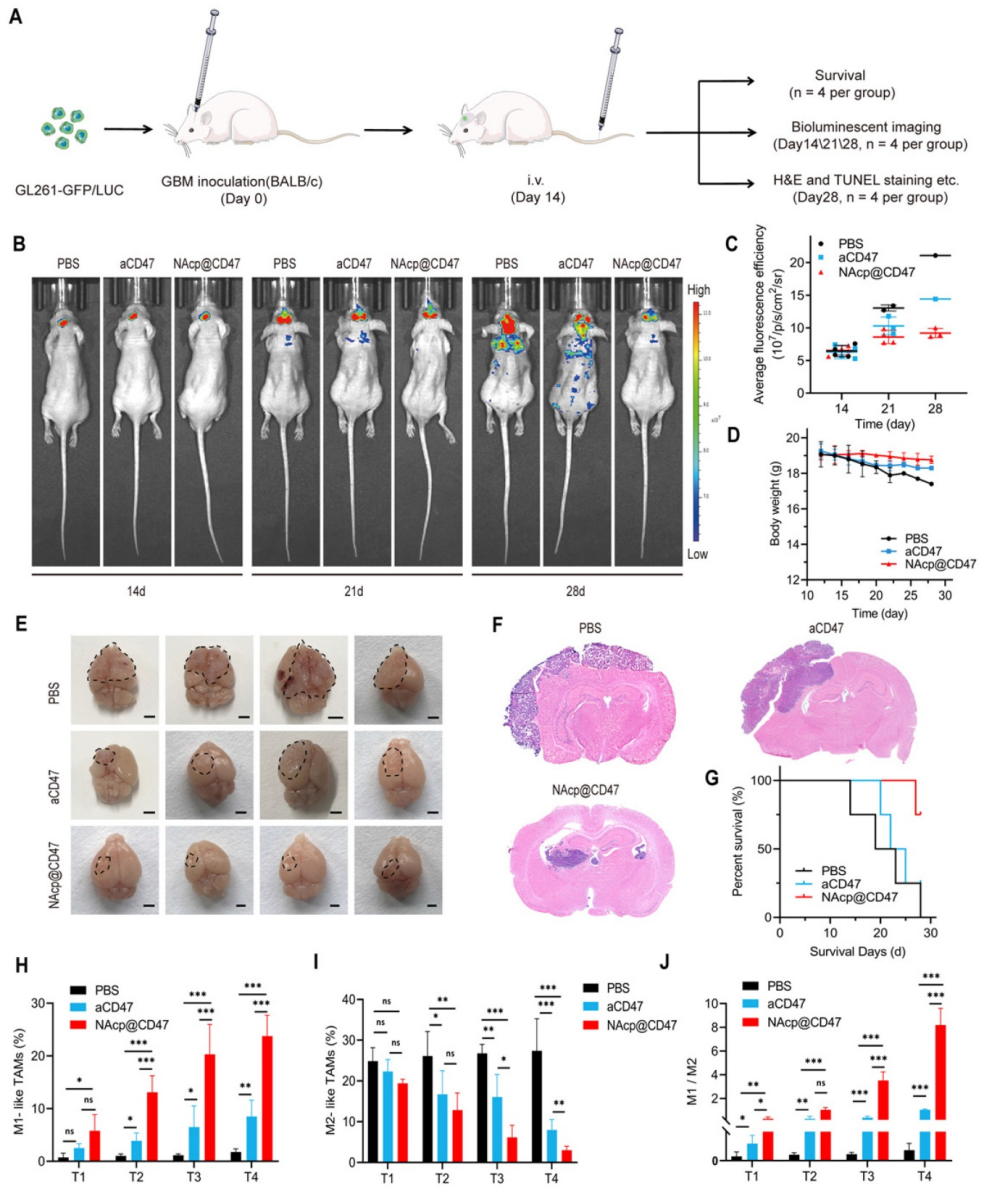
NAcp@CD47-mediated therapeutic effects in in vivo orthotopic syngeneic models for GBM. **(A)** Schematic illustrating NAcp@CD47 therapy in orthotopic syngeneic models for brain tumors with stereotaxic injection of GL261-GFP/LUC cells. **(B-C)** Imaging of in vivo bioluminescence in GL261 tumors in response to treatment with PBS, aCD47 control, and NAcp@CD47 on day 14, 21, and 28 after injection, as detected using an IVIS spectrum instrument (B) and by relative quantification (C). **(D)** Weight changes of mice in different groups. **(E)** Photographic illustration of brains with syngeneic tumors harvested from mice intravenously injected with PBS, aCD47 control, and NAcp@CD47 at the end point (n = 4). Scale bar, 1 mm. **(F)** Paraffin-embedded brain tissue sections in different groups subjected to H&E immunohistochemical staining. Scale bar, 200 mm. **(G)** Kaplan-Meier survival curves of mice in different groups. **(H)** Changes in the percentages of M1-like TAMs during therapeutic process. **(I)** Changes in the percentages of M2-like TAMs from tumor tissues during therapeutic process. **(J)** Changes in the ratio of M1/M2 from tumor tissues during therapeutic process. *P < 0.05; **P < 0.01; ***P < 0.001; ns, not significant.

The schematic of the treatment scheme (Figure 4A) depicts that the fourteen days after stereotaxic injection of GL261-GFP/LUC cells, aCD47 antibody monotherapy and NAcp@CD47 were injected intravenously into mice through the tail vein every 2.5 days for a total of seven injections. Tumor growth was monitored and verified by using GFP fluorescence IVIS spectrum instrument (488/507 nm) (Figure 4B). The existence of GFP could be more likely to reflect the actual size of the mouse brain tumor and eliminate the influence from luciferase substrate. All BALB/c mice were epilated with depilatory cream after anesthesia to minimize background fluorescence. Mice treated with NAcp@CD47 displayed obviously improved control of tumor growth as compared to control groups on day 28, which was only observed restrictedly in the brain (Figure 4C). The body weight of mice in distinct groups was not affected by the treatment (Figure 4D). In addition, no pathological differences in blood parameters and H&E staining of histological sections from the heart, liver, spleen, lung, and kidney between the NAcp@CD47-treated group and control group were observed (Figure S13 and S14), illustrating the well-tolerated biosafety of NAcp@CD47 in vivo. We evaluated the liver functions of mice using blood biochemical analysis. As shown in Table S1, there were no significant differences in these six parameters between NAcp@CD47 and control group, which indicated that NAcp@CD47 did not impair liver function. The fluorescence signals were reduced gradually in the blood of mice after intravenous injection of NAcp@CD47 (Figure S15), while ex vivo fluorescence imaging showed no significant differences in the heart, liver, spleen, lung, and kidney between the NAcp@CD47-treated group and the control group (Figure S16). After the treatments were completed, harvested brains with syngeneic tumors in distinct groups were compared (Figure 4E), revealing effective anti-glioma efficacy of tumor growth in NAcp@CD47-treated group as compared to controls analyzed using H&E staining (Figure 4F). An increased survival benefit was also observed in mice treated with NAcp@CD47 compared with controls (Figure 4G). To determine the potential immune changes in the brains of mouse orthotopic models during therapeutic process, we collected and analyzed the tumor tissues at 4 points in time when the mice were still alive based on the Kaplan-Meier survival curves. At day 1 (T1), day 4 (T2), day 7 (T3), and day 10 (T4) after tail intravenous injection, tumor tissues were harvested and digested using collagenase for flow cytometric analysis (Figure S17). We quantified the TAM polarization inside the tumor by labeling microglia and a small number of bone marrow macrophages with CD86 (proinflammatory M1-like TAMs) and CD206 (anti-inflammatory M2-like TAMs) among the CD11b^+^F4/80^+^ cells. Interestingly, during the therapeutic process, the percentage of M1-like TAMs was significantly increased (Figure 4H), while the percentage of M2-like TAMs was dramatically decreased (Figure 4I), and the ratio of M1/M2 was markedly elevated (Figure 4J). These data support that NAcp@CD47 can reprogram microglia and macrophages from M2 toward M1, and the effects of NAcp@CD47 on reprogramming are superior to controls.

### NAcp@CD47-mediated antitumor immune response in in vivo orthotopic syngeneic models for GBM

**Figure 5:**
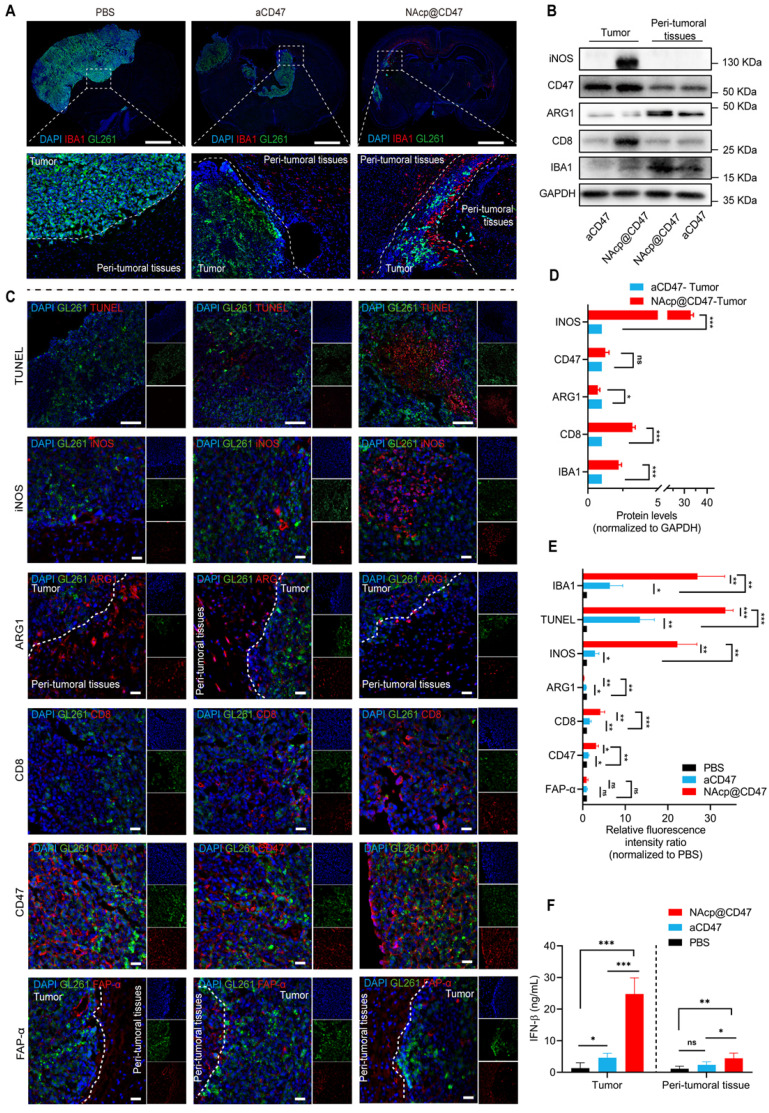
NAcp@CD47-mediated antitumor immune response in in vivo orthotopic syngeneic models for GBM. **(A)** Immunofluorescence analysis of frozen sections stained with IBA1 (red) in GBM tumors and peri-tumoral tissues in different groups. Scale bar, 200 mm. **(B, D)** iNOS, CD47, ARG1, CD8, and IBA1 protein expression levels in GBM tumors and peri-tumoral tissues of groups treated with NAcp@CD47 and aCD47, as determined by western blotting (B) and relative quantification (D). **(C, E)** Representative fluorescence microscopic images of frozen sections stained with TUNEL, iNOS, CD47, ARG1, CD8, CD47 and FAP-α in different groups (C) and relative quantification of the staining intensity (E). **(F)** The levels of IFN-β in tumor tissues and peri-tumoral tissues by ELISA. Scale bar, 200 ìm. *P < 0.05; **P < 0.01; ***P < 0.001; ns, not significant.

To further evaluate whether NAcp@CD47 activated the immune response in orthotopic syngeneic models, we detected a variety of phagocytosis markers expressed in TAMs 49. In the group treated with NAcp@CD47, we observed higher number of microglia in and migrating towards tumor tissues and peri-tumoral tissues analyzed by ionized calcium-binding adapter molecule 1 (IBA) (Figure 5A). M1-like microglia were characterized by the M1-specific markers CD86 and iNOS. M2-like microglia were characterized by the M2-specific markers CD206 and ARG1 57. In the western blotting analysis and relative quantification, compared with those of the control group treated with CD47 antibody monotherapy, the excised tumors of NAcp@CD47-treated mice showed obvious upregulation of iNOS, CD8, and IBA1 protein expression levels and downregulation of ARG1 protein expression levels (Figure 5B, D). The combined treatment significantly increased GBM cell phagocytosis by microglia compared with that obtained by aCD47 treatment alone. Further tissue immunofluorescence analysis of pericarcinomatous tissues revealed that compared with aCD47, NAcp@CD47 significantly increased both the number of infiltrating activated microglial cells and the depth of tissue infiltration, as indicated by IBA1 expression (Figure S18). The harvested samples were also analyzed by immunofluorescence staining, and the data indicated that the expression of IBA1, TUNEL, INOS, CD47, and CD8 was significantly increased while that of ARG1 was significantly decreased in the NAcp@CD47-treated group compared with that in control groups (Figure 5C, E). The expression of FAP-α in tumor tissues and peri-tumoral tissues were also detected (Figure 5F). The above findings indicated that NAcp@CD47 could reprogram microglia and macrophages from an anti-inflammatory M2-phenotype to a pro-inflammatory M1-phenotype. The levels of IFN-β in tumor tissues and peri-tumoral tissues were assessed using ELISA. The results showed that NAcp@CD47 activated STING pathway and increased IFN-β levels compared with controls. Importantly, there were no apparent side effects in the animal model. The potential problems associated with the use of anti-CD47 antibodies as anti-cancer treatments include possible off-target effects that may cause anemia. To alleviate this side-effect, this study designed smart response nanocapsules, NAcp@CD47, to specifically release antibody drugs at the tumor site to avoid damage to normal cells, while still being able to increase tumor cell removal rate 58. In summary, these findings suggest that NAcp@CD47 can effectively penetrate the BBB, activate macrophages/microglia and immune responses, and inhibit the growth of brain tumors in orthotopic models. The present strategy thus provides an attractive approach for the treatment of GBM 23.

## Conclusions

In conclusion, we have successfully developed novel BBB-permeable nanocapsules acting as “Trojan horse”, NAcp@CD47, that enable the effective delivery of therapeutic cargoes to target GBM for immunotherapy. Both in vitro and in vivo studies demonstrated that NAcp@CD47 could increase the polarization of M1-phenotype TAMs, help reduce tumor immunosuppression, and inhibit tumor growth by phagocytosis of macrophages and microglia. Our findings suggest that NAcp@CD47 reshaped the immune microenvironment by switching the phenotype of microglia and macrophages to synergistically enhance phagocytosis, and this approach is a promising strategy for the immunotherapy of GBM. More importantly, this study provides the basis for a biomimetic strategy to overcome biological barriers in vivo, and thus improve the diagnosis and treatment of CNS diseases.

## Supplementary Material

Supplementary figures and table.
