# Supplementary material for: Co-delivery of phagocytosis checkpoint and STING agonist by a Trojan horse nanocapsule for orthotopic glioma immunotherapy
Source: Theranostics. 2022 Jul 18;12(12):5488–503. doi: 10.7150/thno.73104 (PMC9330518; doi:10.7150/thno.73104)
Supplement: Supplementary file 1 — Supplementary figures and table. [file thnov12p5488s1.pdf]

Supplementary Material

Co-delivery of phagocytosis checkpoint and STING agonist by A Trojan horse nanocapsule for orthotopic glioma immunotherapy

Ying Zhou[1,2]†, Yuxin Guo[2]†, Lufei Chen[1]†, Xiaoli Zhang[1]†, Wei Wu[1], Zhimin Yang[1,2], Xuejie Li[1,2], Yuanzhuo Wang[2,3], Zhiyuan Hu[1,2,3,4]*, Zihua Wang[1,2]*

[1] Fujian Provincial Key Laboratory of Brain Aging and Neurodegenerative Diseases, School of Basic Medical Sciences, Fujian Medical University, Fuzhou, Fujian 350122, China.

[2] CAS Key Laboratory of Standardization and Measurement for Nanotechnology, CAS Key Laboratory for Biomedical Effects of Nanomaterials and Nanosafety, CAS Center for Excellence in Nanoscience, National Center for Nanoscience and Technology, Beijing 100190, China.

[3] School of Nanoscience and Technology, Sino-Danish College, University of Chinese Academy of Sciences, Beijing 100049, China.

[4] School of Chemical Engineering and Pharmacy, Wuhan Institute of Technology, Wuhan, 430205, China.

†These authors contributed equally to this work.
*Corresponding author. Email: wangzh@fjmu.edu.cn; huzy@nanoctr.cn

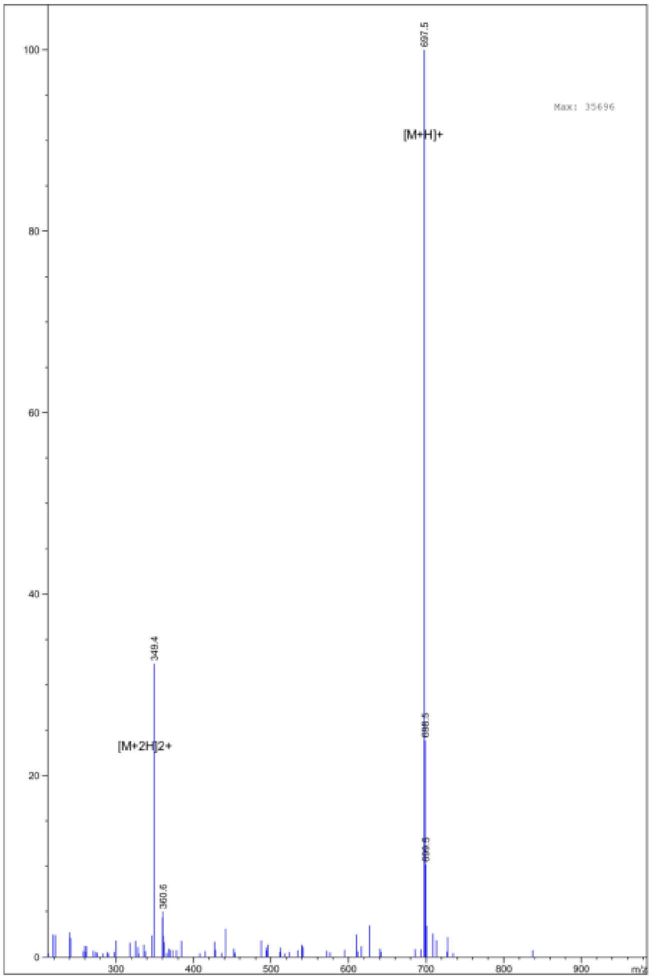

Figure S1. The biacrylated FAPα-responsive peptide crosslinker by ESI-MS.

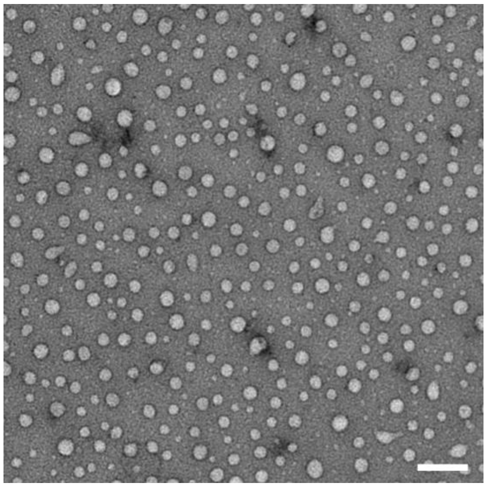

Figure S2. TEM images of NAcp without cargoes (scale bar, 200 nm).

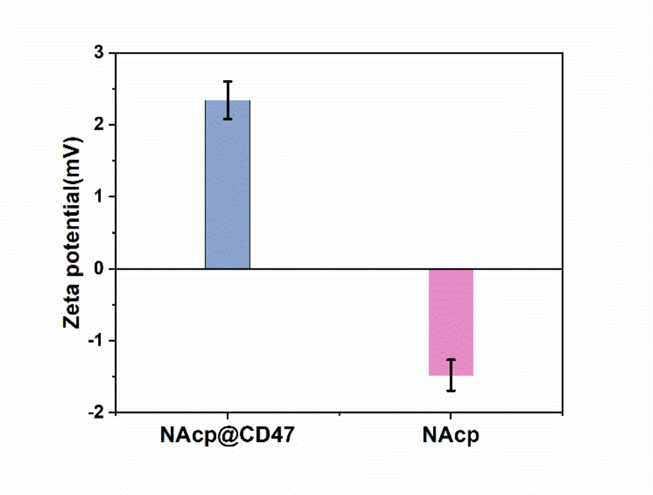

Figure S3. Zeta potential of NAcp@CD47 and NAcp.

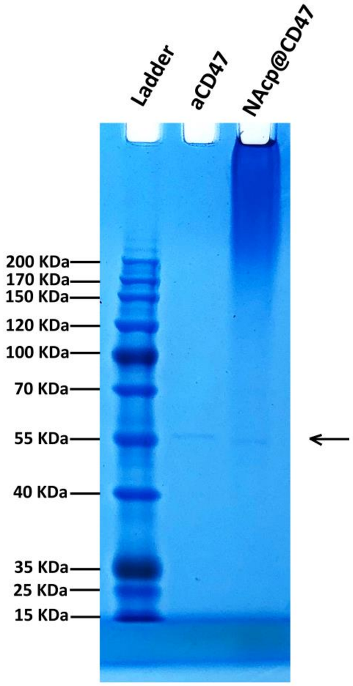

Figure S4. Coomassie brilliant blue (CBB) staining of CD47 antibody and CD47@NAcp in SDS-polyacrylamide gel electrophoresis (PAGE).

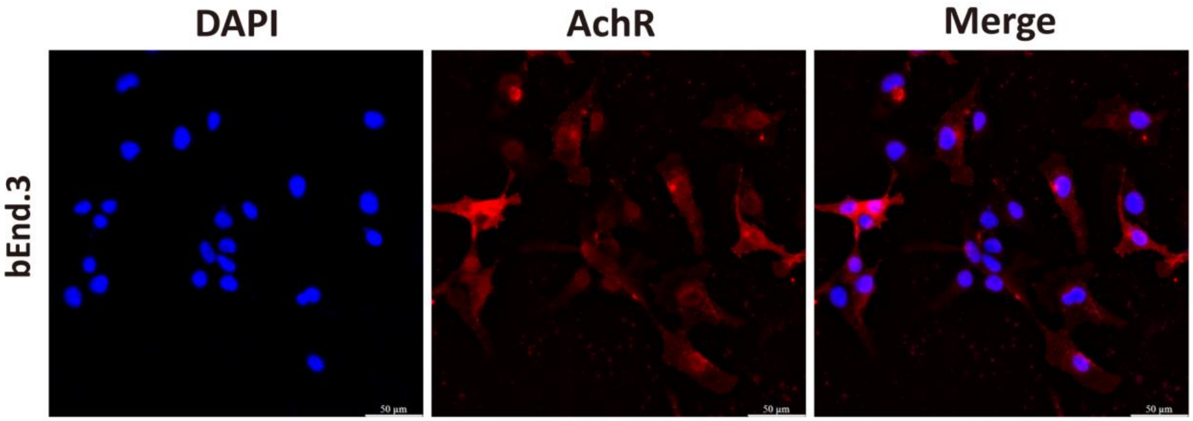

Figure S5. The expression of nAChRs on mouse brain capillary endothelial bEnd.3 cells analyzed by immunofluorescence.

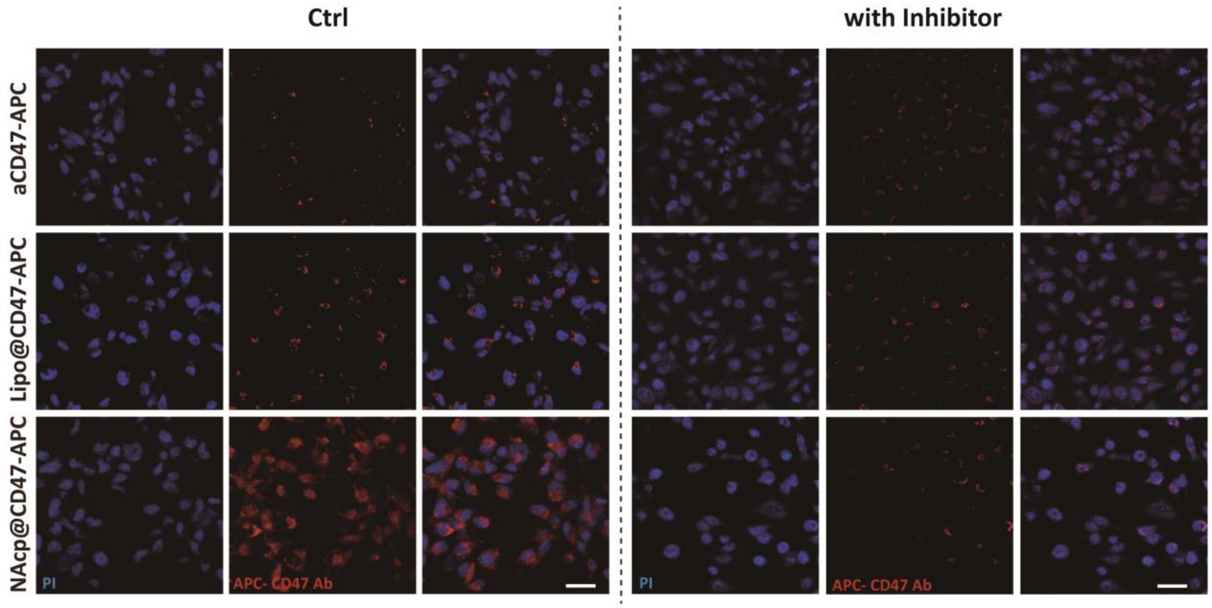

Figure S6. Representative fluorescence images of GL261 cells in the in vitro BBB model after 4 h of incubation with NAcp@CD47, Lipo@CD47 or controls labeled with APC with or without nAChR inhibitor. Scale bar, 20 µm. n=3.

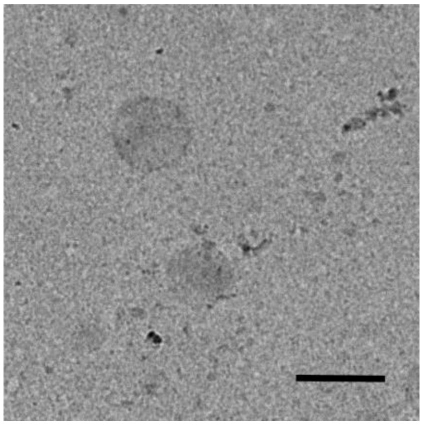

Figure S7. The morphology of liposomes detected by TEM. Scale bar, 200 nm.

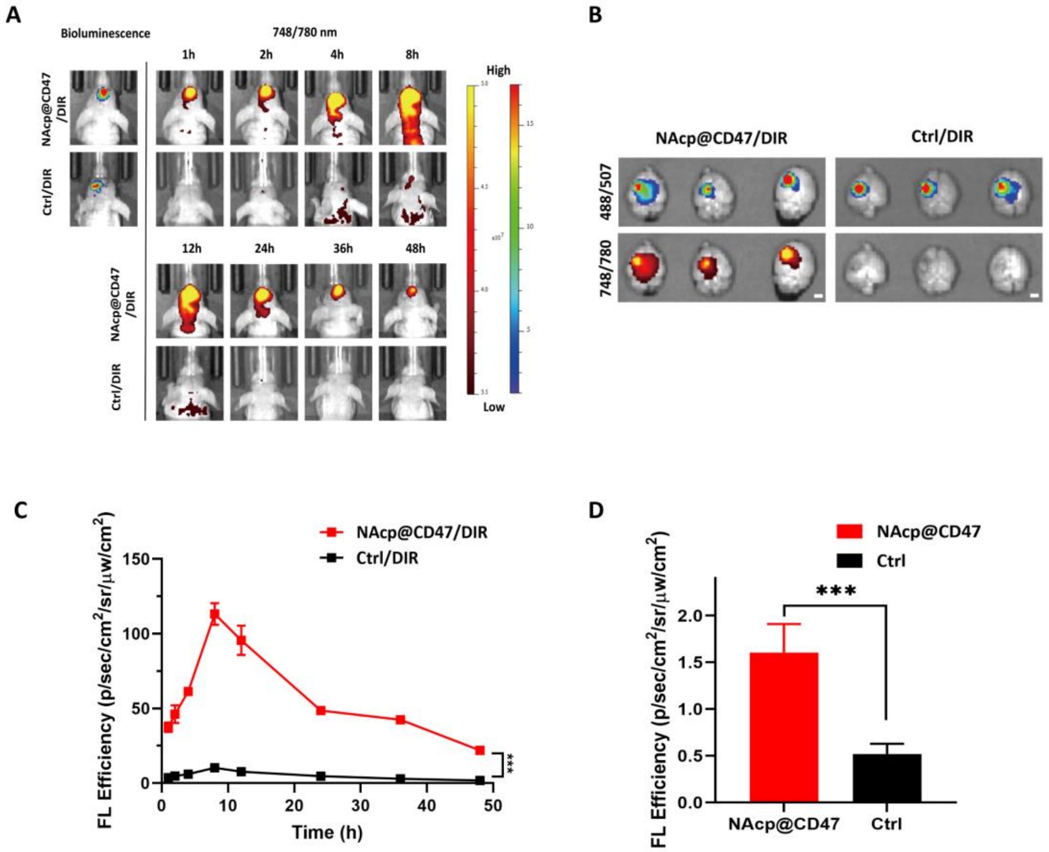

Figure S8. In vivo fluorescence imaging of mice at indicated time points after intravenously injected with NAcp@CD47/DiR or Ctrl/DiR control group. **(**A) In vivo fluorescence imaging of mice at indicated time points after intravenously injected with NAcp@CD47-DiR or DiR control group. (B) Ex vivo fluorescence imaging of brains from mice after injection of NAcp@CD47 or control group. Excitation/emission wavelengths 488/507 nm for GFP and excitation/emission wavelengths 748/780 nm for DiR revealed a colocalization of GBM and NAcp@CD47 (scale bar, 2 mm). (C, D) Quantitative analysis of in vivo (C) and ex vivo (D) fluorescence efficiency of NAcp@CD47 and control group in the brain ($n = 3$). ***$P < 0.001$.

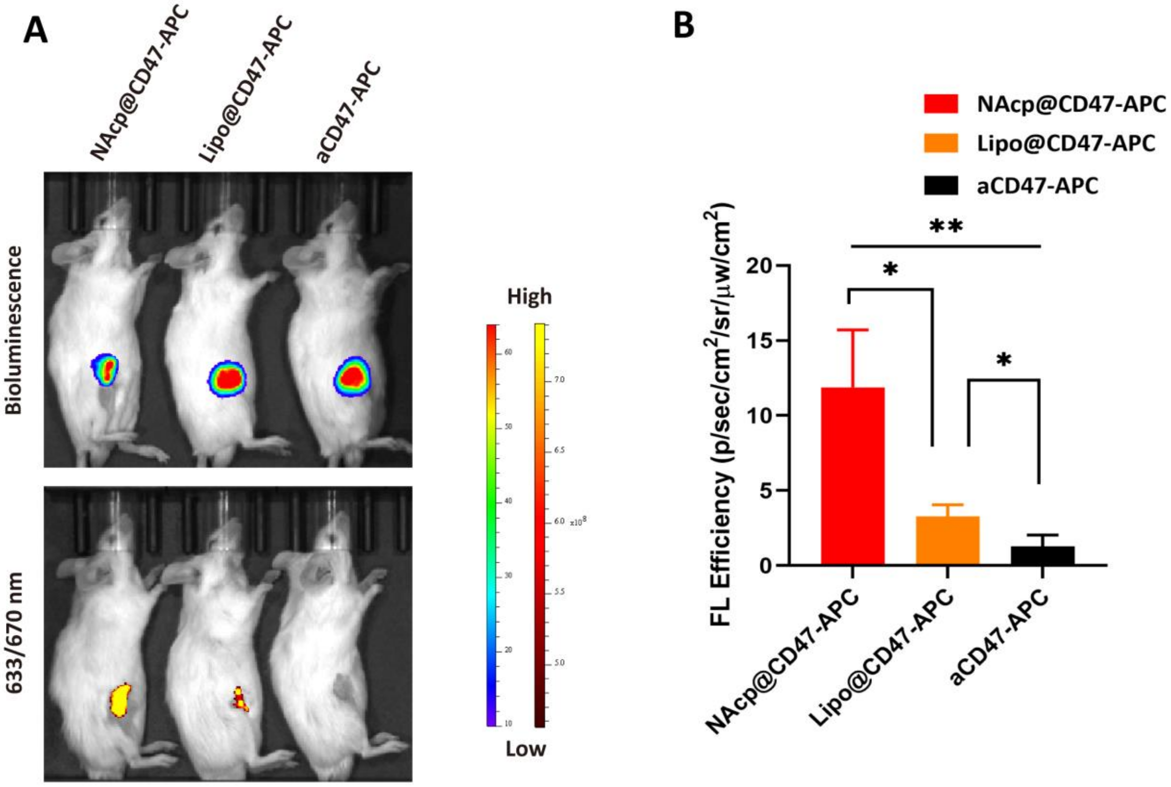

Figure S9. The targeting effects of NAcp@CD47-APC, Lipo@CD47-APC, or aCD47-APC in subcutaneous implanted tumor model mice. (A) In vivo fluorescence imaging of NAcp@CD47-APC, Lipo@CD47-APC, or aCD47-APC in subcutaneous implanted tumor model mice via tail vein injection. (B) Quantitative analysis of fluorescence efficiency of each group (n = 3). $^{*}P < 0.05$, $^{**}P < 0.01$.

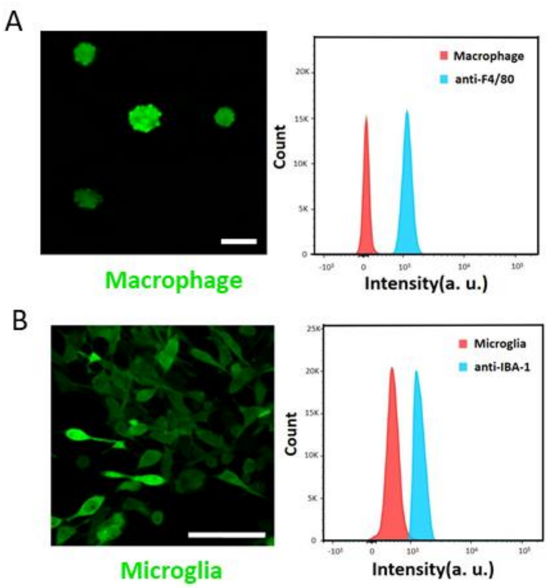

Figure S10. Representative fluorescence images and flow cytometric analysis images of peritoneal macrophage (A) and brain microglia (B) from eGFP transgenic mice. Scale bar, 200 µm.

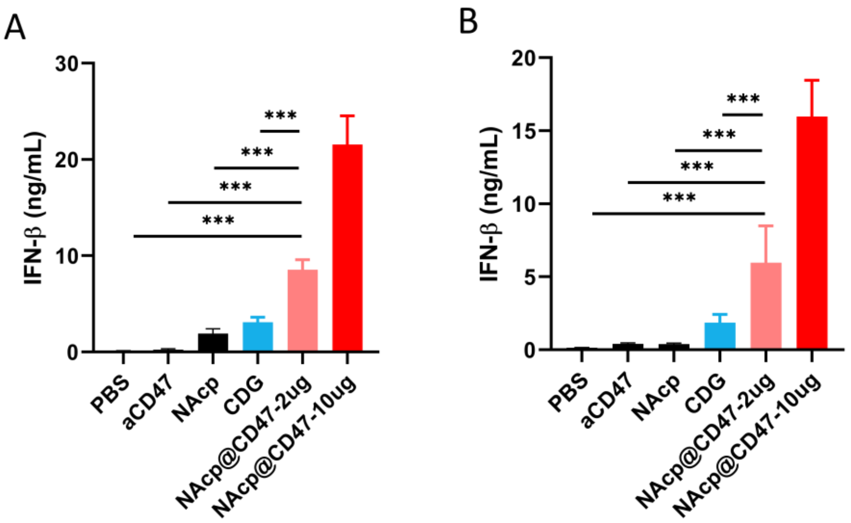

Figure S11. The secretion of IFN-β in BV2 cells (A) and RAW 264.7 cells (B) treated with NAcp@CD47 or other controls analyzed by ELISA assays.

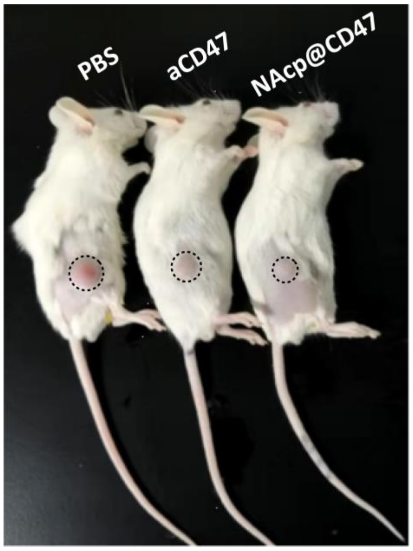

Figure S12. Representative photographic illustration of syngeneic GBM models intravenously injected with PBS, CD47 antibody control, and NAcp@CD47 respectively at day 28.

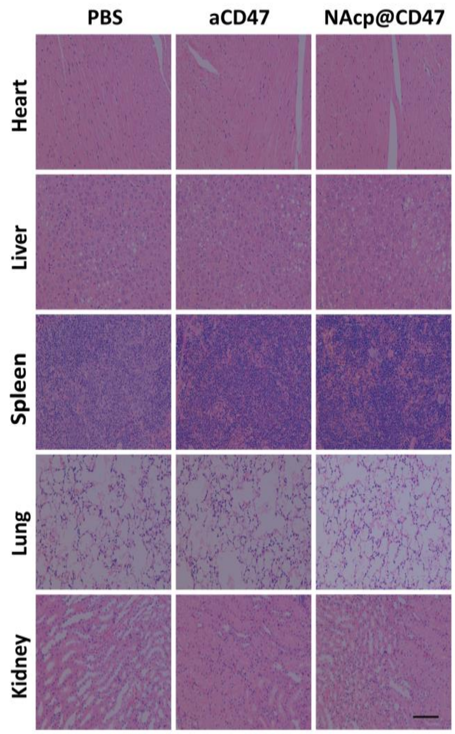

Figure S13. Paraffin-embedded immunohistochemical tissue sections in heart, liver, spleen, lung, and kidney from mice treated with PBS, CD47 antibody control, and NAcp@CD47 respectively at day 28 stained with H&E. Scale bar, 200 μm.

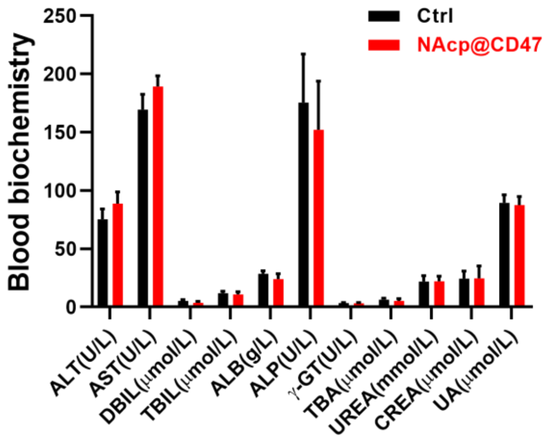

Figure S14. Comparison of selected serum biochemical parameters including alanine amino transferase (ALT), aspartate amino transferase (AST), direct bilirubin (DBIL), total bilirubin (TBIL), albumin (ALB), alkaline phosphatase (ALP), gamma-glutamyltransferase (γ-GT), total bile acid (TBA), urea (UREA), creatinine (CREA), and uric acid (UA) between mice intravenously injected with NAcp@CD47 or PBS as controls through the tail vein.

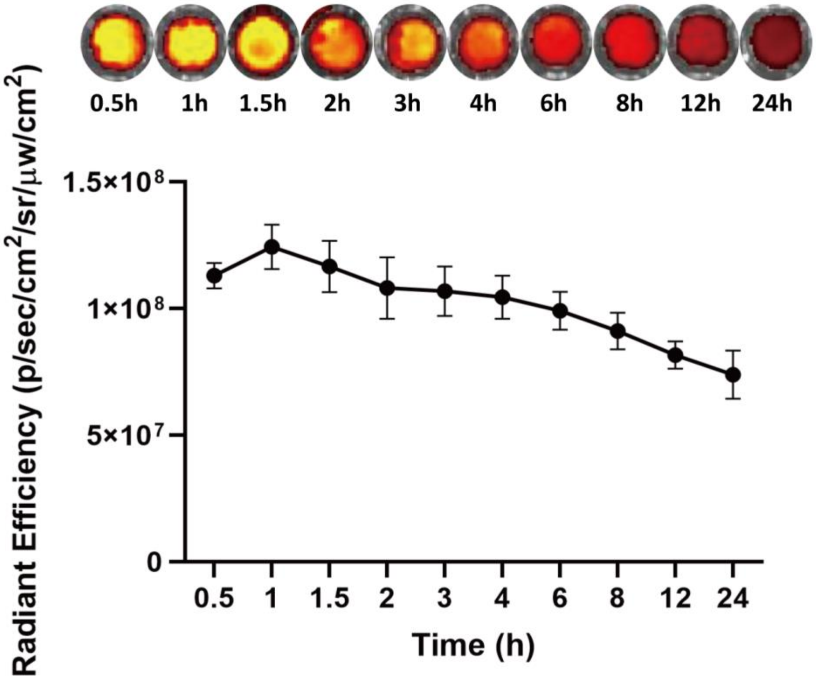

Figure S15. Quantitative analysis of the fluorescence signals at different time points in the blood of mice after intravenous injection of NAcp@CD47 labeled with 1,1-dioctadecyl-3,3,3,3-tetramethylindotricarbocyanine iodide (DiR) through the tail vein.

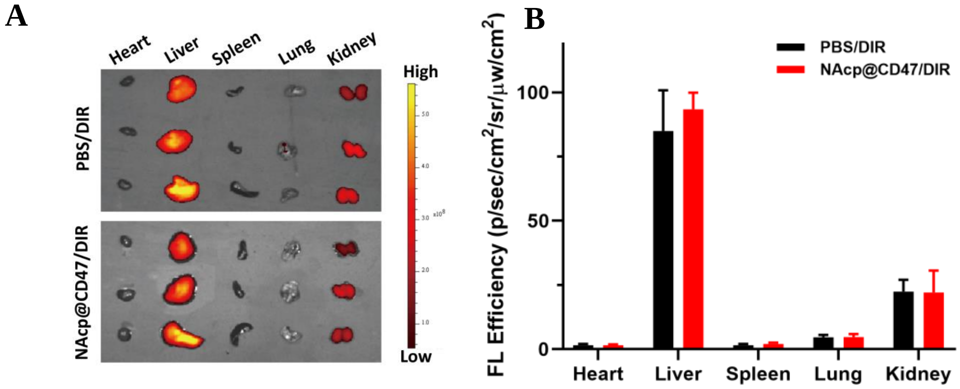

Figure S16. Ex vivo fluorescence imaging (A) and quantitative analysis of fluorescence efficiency (B) in mouse heart, liver, spleen, lung, and kidney 48 h after intravenous injection of NAcp@CD47 or control group.

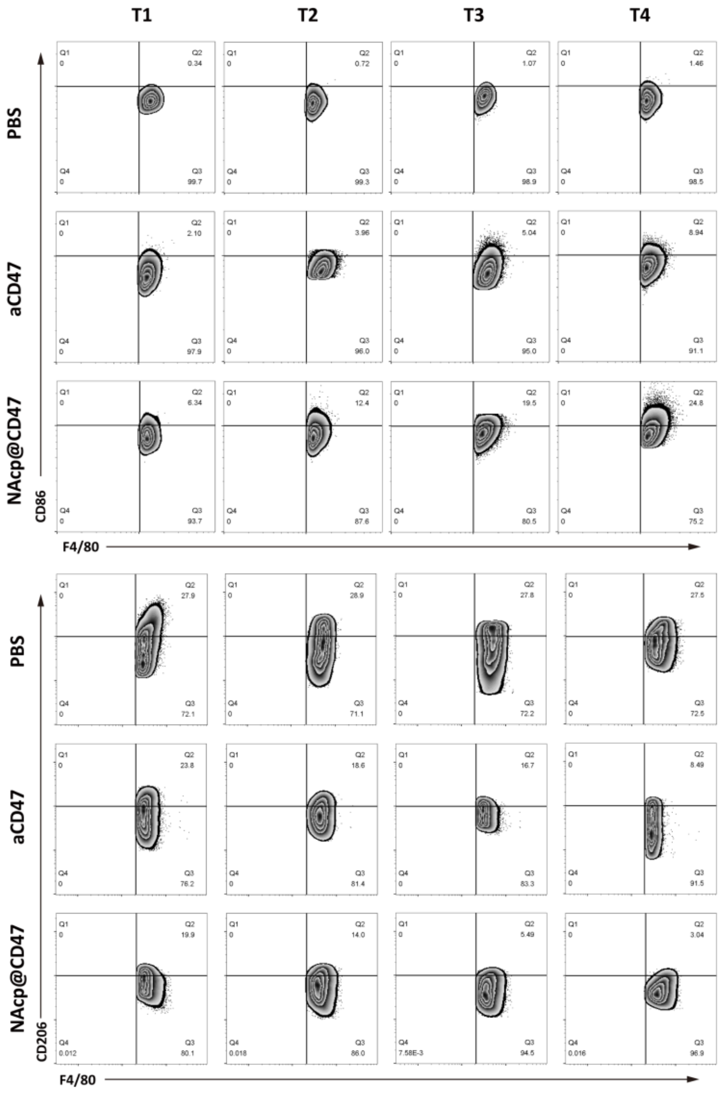

Figure S17. Flow cytometry analysis of tumor tissues harvested and digested using collagenase at day 1 (T1), day 4 (T2), day 7 (T3), and day 10 (T4) after tail intravenous injection.

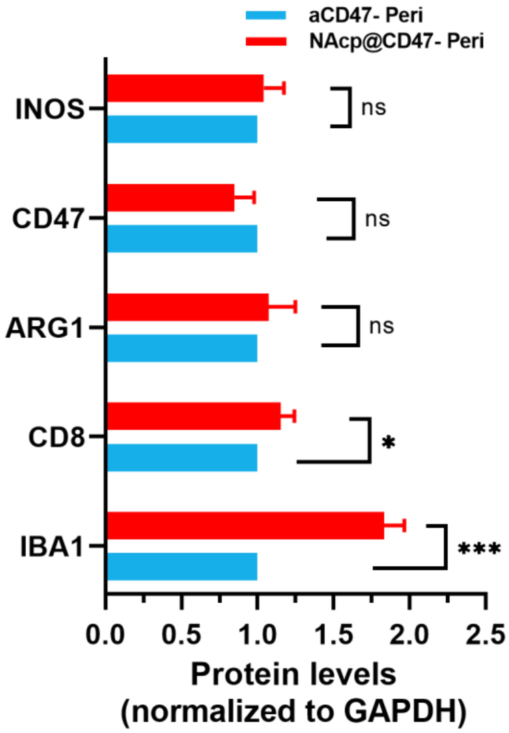

Figure S18. NAcp@CD47-mediated antitumor immune response in peri-tumoral tissues of orthotopic syngeneic models for GBM in vivo. iNOS, CD47, ARG1, CD8, and IBA1 protein expression levels in peri-tumoral tissues of groups treated with NAcp@CD47 and aCD47 analyzed by western blotting. *$P < 0.05$; ***$P < 0.001$; ns, not significant.

Table S1. Comparison of complete blood count between mice intravenously injected with NAcp@CD47 or PBS as controls through the tail vein.

Parameters	Abbreviation	Units	Reference range	Ctrl	NAcp@CD47
White blood cell number	WBC	10^9/L	0.8-6.8	2.4	3.3
Lymphocyte number	Lymph#	10^9/L	0.7-5.7	2.1	2.9
Monocyte number	Mon#	10^9/L	0.0-0.3	0.1	0.1
Neutrophilic granulocyte number	Gran#	10^9/L	0.1-1.8	0.2	0.3
Lymphocyte percentage	Lymph%	%	55.8-90.6	87.8	88.0
Monocyte percentage	Mon%	%	1.8-6.0	2.1	2.6
Neutrophilic granulocyte percentage	Gran%	%	8.6-38.9	10.1	9.4
Red blood cell number	RBC	10^12/L	6.36-9.42	6.81	7.35
Hemoglobin	HGB	g/L	110-143	117	112
Hematocrit	HCT	%	34.6-44.6	39.6	32.2
Mean corpuscular volume	MCV	fL	48.2-58.3	43.6	43.9
Mean corpuscular hemoglobin	MCH	pg	15.8-19	17.1	15.2
Mean corpuscular hemoglobin concentration	MCHC	g/L	302-353	345	347
Red blood cell volume distribution width	RDW	%	13-17	15.7	16.7
Blood platelet count	PLT	10^9/L	450-1590	579	545
Mean platelet volume	MPV	fL	3.8-6.0	6.7	5.3
Platelet distribution width	PDW			16.9	17.0
Plateletcrit	PCT	%		0.052	0.076
